# Review of Diagnostic Biomarkers in Autoimmune Pancreatitis: Where Are We Now?

**DOI:** 10.3390/diagnostics11050770

**Published:** 2021-04-25

**Authors:** Masataka Yokode, Masahiro Shiokawa, Yuzo Kodama

**Affiliations:** 1 Department of Gastroenterology and Hepatology, Kyoto University Graduate School of Medicine, Kyoto 606-8507, Japan; y0428@kuhp.kyoto-u.ac.jp; 2 Division of Gastroenterology, Department of Internal Medicine, Kobe University Graduate School of Medicine, Kobe 650-0017, Japan; kodama@med.kobe-u.ac.jp

**Keywords:** autoimmune pancreatitis, IgG4-related disease, autoantibody, biomarker, diagnosis

## Abstract

Autoimmune pancreatitis (AIP) is a pancreatic manifestation of an IgG4-related disease (IgG4-RD). AIP lacks disease-specific biomarkers, and therefore, it is difficult to distinguish AIP from malignancies, especially pancreatic cancer. In this review, we have summarized the latest findings on potential diagnostic biomarkers for AIP. Many investigations have been conducted, but no specific biomarkers for AIP are identified. Therefore, further studies are required to identify accurate diagnostic biomarkers for AIP.

## 1. Introduction

Autoimmune pancreatitis (AIP) has recently been recognized as a distinct form of chronic pancreatitis with segmental or diffuse enlargement of the pancreas and irregular narrowing of the main pancreatic duct [1]. The concept of AIP was first proposed by Yoshida et al. in 1995 through a case report on steroid-responsive pancreatitis [2]. In 2001, Hamano et al. reported elevated serum IgG4 levels in more than 90% of Japanese patients with AIP, suggesting a relationship between AIP and IgG4 [3]. In addition to laboratory findings, IgG4 can also be useful in the pathological diagnosis of AIP because of the tissue infiltration by IgG4-positive plasma cells. In 2003, Kamisawa et al. reported severe or moderate infiltration of IgG4-positive plasma cells in other organs, such as the bile duct, gallbladder, and salivary glands, and proposed that AIP is a lesion of an IgG4-related systemic autoimmune disease [4]. Furthermore, several concepts regarding systemic disease related to IgG4 have been proposed by other groups. In 2006, Yamamoto et al. proposed that Mikulicz’s disease, which was previously thought to be an atypical type of Sjögren’s syndrome, can be newly classified as an “IgG4-related plasmacytic syndrome” [5]. Masaki et al. proposed that the new clinical entity was a lymphoproliferative disease and named it “IgG4 multiorgan lymphoproliferative syndrome” [6]. Umehara et al. unified these concepts in 2011 as IgG4-related disease (IgG4-RD), and AIP was classified as one of the manifestations of IgG4-RD [7].

AIP can be sub-classified into two subtypes based on the clinical and pathological features: type 1 AIP and type 2 AIP [8]. Type 1 AIP is characterized by high serum IgG4 levels and increased IgG4-positive plasma cells in the tissues. In comparison, type 2 AIP is not associated with IgG4 and has the histological features of granulocytic epithelial lesions in the pancreatic ducts [8]. Type 1 AIP is considered a pancreatic lesion of IgG4-RD [9]. AIP, similar to other forms of IgG4-RD, shows a remarkable response to steroid therapy, which suggests that this disease may be associated with an autoimmune mechanism [1,10]. In addition, AIP responds to the anti-CD20 antibody, rituximab, and this supports the association with autoimmunity [11]. AIP often relapses, even after successful remission following steroid therapy [12,13]. Therefore, maintenance steroid therapy (MST) could be useful for preventing AIP relapse [12,13].

Elevation of serum IgG4 levels is not specific for the diagnosis of AIP because this happens in a variety of diseases, including cancers, infections, and other autoimmune diseases [14]. It is essential to differentiate between AIP and pancreatic cancer before initiating steroid therapy. Predicting relapse in patients with AIP is also important, considering the high rate of AIP relapse. We previously identified laminin 511-E8 as a pathogenic antigen for AIP [15] and suggested that anti-laminin 511-E8 may be a useful biomarker for AIP. However, various studies, including ours, could not identify specific biomarkers for the diagnosis of AIP (Table 1). In this review, we summarize the current findings regarding the potential biomarkers for AIP and discuss a new candidate biomarker for IgG4-RD.

## 2. Potential Biomarkers for Diagnosis of AIP

### 2.1. IgG4

**Figure 1:**
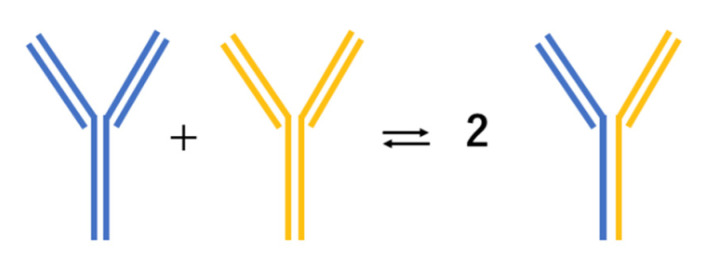
Unique mechanism of IgG4 antibodies becoming bispecific via Fab-arm exchange.

IgG4 is the least common among the four subclasses of IgG, accounting for only about 4% of total IgG in normal serum [27]. IgG4 antibodies form bi-specific antibodies through Fab-arm exchange, functioning as monovalent antibodies (Figure 1) [28]. These modified antibodies can bind to two different antigens; however, they are unable to activate the classical complement system. In addition, these asymmetric antibodies cannot form immune complexes. These characteristics suggest that IgG4 antibodies are more likely to be anti-inflammatory than pro-inflammatory [28].

To differentiate between AIP and pancreatic cancer, Hamano et al. proposed the use of a cutoff value of 135 mg/dL for serum IgG4 concentrations, which provided a sensitivity of 95% (19/20) and specificity of 97.1% (68/70) [3]. However, Ghazale et al. studied 510 patients, including 45 with AIP, and reported that an elevated serum IgG4 level > 140 mg/dL had a sensitivity of only 75.6% (34/45) and a specificity of 93.1% (433/465). In addition, 9.6% (13/135) of the patients with pancreatic cancer had elevated serum IgG4 levels [16]. IgG4 levels are not useful for the diagnosis of type 2 AIP [8]. Therefore, elevated IgG4 cannot be used as the sole marker for the diagnosis of AIP. Recently, Shih et al. analyzed the serum IgG-glycosylation profiles of AIP and PDAC patients, using liquid chromatography–electrospray ionization mass spectrometry [29]. Classification and regression tree analysis revealed that galactosylation ratios and sialylation ratios of IgG2 and IgG4 are useful in differentiating between AIP and PDAC, with high accuracy [29]. Therefore, combining IgG4 with other biomarkers could be useful for the diagnosis of AIP.

### 2.2. Antibodies to Carbonic Anhydrase II

Carbonic anhydrase II (CA II) is expressed in the ductal epithelial cells of the exocrine organs, such as the pancreas, salivary gland, bile duct, and kidney [30,31]. Kino-Ohsaki et al. investigated the presence of autoantibodies to CA II in patients with AIP [17]. An enzyme-linked immunosorbent assay (ELISA) was performed using the sera of 74 patients with chronic pancreatitis. Increased serum CA II antibody was present in 33.3% (11/33) of the patients with AIP, compared to the presence in 15% (3/20) of patients with alcoholic pancreatitis and 0% (0/7) of patients with cholecystic pancreatitis. However, increased CA II antibody was detected in 61.9% (13/21) of patients with Sjögren’s syndrome [17]. The percentage of patients with AIP that have increased serum CA II antibody is lower than that of patients with Sjögren’s syndrome [32]. Taken together, these findings indicate that the autoantibody to CA II lacks disease specificity for the diagnosis of AIP.

### 2.3. Antibodies to Lactoferrin

Lactoferrin (LF) is an iron-binding protein usually found at a low concentration in pancreatic juice, but its secretion tends to increase in individuals with chronic pancreatitis [33]. Okazaki et al. reported that antibodies to LF are detected using ELISA in 76.5% (13/17) of patients with AIP [18]. The level of serum antibodies to LF is significantly higher in patients with AIP compared to that in patients with gallstone-related pancreatitis, patients with alcoholic chronic pancreatitis, and controls. However, antibodies against LF are also found in individuals with other immune diseases, such as ulcerative colitis and primary sclerosing cholangitis (PSC), and therefore, are considered to be less disease-specific than those against CA II [18,34].

### 2.4. Antibodies to Pancreatic Secretory Trypsin Inhibitor

Pancreatic secretory trypsin inhibitor (PSTI) is produced in pancreatic acinar cells and is present along with trypsinogen as zymogen granules [35,36]. PSTI suppresses approximately 20% of trypsin activity, thereby preventing pancreatitis [37]. Asada et al. identified PSTI as a potential disease-specific antibody for AIP [19]. A complementary DNA (cDNA) library was immunoscreened using sera from patients with AIP. The entire coding sequence of the PSTI cDNA was present in the positive clones. The investigators hypothesized that PSTI could be a target antigen in AIP because mutations in PSTI are closely related to the development of hereditary pancreatitis and idiopathic chronic pancreatitis [19]. Recombinant PSTI proteins were produced and used as antigens in ELISA and Western blot analyses. Antibodies to PSTI were detected through Western blotting in 42.3% (11/26) of patients with AIP and using ELISA in 30.8% (8/26) of the patients. In contrast, the sera of all the other patients tested were negative for antibodies to PSTI in both Western blotting and ELISA [19]. These investigators also examined the immune responses of mice injected with polyinosinic polycytidylic acid (poly I:C), which induces pancreatitis [38]. Notably, 91.7% of poly I:C-injected mice tested positive for antibodies to PSTI. The frequency of antibodies against PSTI detected in poly I: C-injected mice are higher than that of antibodies against CA II (33.3%) and LF (45.8%). The epitope of the anti-PSTI antibodies is at the site of PSTI involved in the trypsin-inhibiting activity [38]. It is possible that anti-PSTI antibodies suppress the action of PSTI, leading to excessive trypsin activity in the pancreas [19,37,38]. However, whether the antibodies to PSTI are the actual pathogenic autoantibodies of AIP remains to be elucidated.

### 2.5. Antibodies to Amylase-2A and Heat-Shock Protein 10

Endo et al. have considered amylase-2A as a specific biomarker, not only for AIP but also for fulminant type 1 diabetes (FT1DM) [20]. They screened a human pancreas cDNA library using sera from patients with AIP. Seven of the 10 positive clones were identical to amylase-2A. Using recombinant amylase-2A, ELISA analysis revealed autoantibodies to amylase-2A in all the patients with AIP, but not in patients with other diseases, such as chronic alcoholic pancreatitis and pancreatic tumor [20]. They also investigated the prevalence of antibodies to amylase-2A in various types of diabetes. Interestingly, 88.2% (15/17) of individuals with FT1DM have antibodies to amylase-2A. The antibody is present in 21.4% (9/42) of patients with acute type 1 diabetes, 6% (4/67) of patients with type 2 diabetes, and 1% (1/100) of controls [20].

This group of researchers also investigated whether patients with AIP had antibodies to heat-shock protein 10 (HSP10) because one of the remaining positive clones was identical to HSP10 [21]. They found that anti-HSP10 antibodies are present in 91.7% (11/12) of patients with AIP and 81.3% (13/16) of patients with FT1DM. However, only 8.3% (2/24) of patients with chronic alcoholic pancreatitis, and 1.4% (1/71) of controls have HSP 10 antibodies [21]. HSP10 maintains mitochondrial function by forming a mitochondrial chaperoning complex with HSP60 [39]. HSP10 is present in the pancreas, especially in acinar cells and islet cells [40]. Further experiments will be required to clarify the association of HSP10 and amylase-2A antibodies with AIP, FT1DM, and other disease such as pancreatic cancer and to determine their potential as biomarkers.

### 2.6. Antibodies to Trypsinogen

Löhr et al. examined RNA and protein expression in pancreatic tissue from patients with AIP and compared them with those from patients with non-AIP chronic pancreatitis [22]. They identified 272 upregulated genes related to immunoglobulins, chemokines, and chemokine receptor production in AIP, and 86 downregulated genes related to pancreatic proteases [22]. Both immunohistochemistry and Western blotting revealed the near absence of trypsin-positive acinar cells. Patients with AIP have high titers of autoantibodies to cationic trypsinogen PRSS1 and anionic trypsinogen PRSS2, but not to mesotrypsinogen PRSS3. In addition, autoantibodies to PSTI are detectable in the sera of patients with AIP [22]. These findings have been confirmed in C57BL6 mice infected with LP-BM5 murine leukemia virus. Based on these findings, Löhr et al. suggested that the loss of acinar cells and production of antibodies to trypsinogens are associated with the pathogenesis of AIP [22]. Further studies are needed to verify whether these antibodies may be useful in the diagnosis of AIP.

### 2.7. Antibodies to Plasminogen-Binding Protein

Frulloni et al. screened a random peptide library using IgG from 20 patients with AIP [23]. Peptide AIP1-7 was detected in the sera of 90% (18/20) of the patients with AIP and 10% (4/40) of patients with pancreatic cancer [23]. As *Helicobacter pylori* infection is associated with the pathogenesis of AIP, and therefore, these researchers compared the sequence of peptide AIP1-7 with known bacterial protein sequences [23,41]. Peptide AIP1-7 shows high homology to plasminogen-binding protein (PBP) of *H. pylori* and to the ubiquitin-protein ligase E3 component, n-recognin 2 [23]. Antibodies to PBP were detected using dissociation-enhanced lanthanide fluorescence immunoassay (DELFIA) in 95% (19/20) of the patients with AIP and 10% (4/40) of the patients with pancreatic cancer. However, no anti-PBP antibodies were detected in patients with alcohol-induced chronic pancreatitis or intraductal papillary mucinous neoplasm [23]. The same group performed a second series of screening and showed that 93.3% (14/15) of patients with AIP, and 1.4% (1/70) of patients with pancreatic cancer had antibodies against the PBP peptide. Additional experiments, including Western blotting and ELISA, confirmed the presence of anti-PBP antibodies in the patients with AIP [23]. In the training and validation groups, antibodies to the PBP peptide were present in 94.3% (33/35) of the patients with AIP but only 4.5% (5/110) of the patients with pancreatic cancer. However, only 53.2% (19/35) of the patients with AIP exhibited elevated serum levels of IgG4, which may suggest a high prevalence of type 2 AIP. The IgG4-positive patients with type 2 AIP were negative for anti-PBP peptide antibodies, while the remaining 16 patients, who were IgG4-negative, had antibodies against the PBP peptide [23]. Further studies are needed to validate the usefulness of these antibodies for the diagnosis of AIP.

### 2.8. Antibodies to Prohibitin

Du et al. evaluated the autoantibody profile of IgG4-RD in multiple cell lines to evaluate candidate autoantigen targets [24], and the cell line HT29 was considered suitable for antigen screening. Western blotting and immunoprecipitation revealed a protein of approximately 30 kDa as a suspected target antigen in IgG4-RD patients. This candidate protein has approximately 40% similarity to human prohibitin [24]. ELISA analysis revealed that antibodies to prohibitin is present in 73.5% (25/34) of patients with definite AIP, 53.3% (8/15) of patients with Mikulicz’s disease, 54.5% (6/11) of patients with retroperitoneal fibrosis, and 89.7% (26/29) of patients with other probable IgG4-RD but in only 1.4% (1/70) of healthy donors [24]. Prohibitin was originally thought to be involved in the inhibition of cell-cycle progression, but recent studies suggest that prohibitin could be essential for the meditation of immune function and inflammation [42,43,44]. Further studies are needed to clarify the pathogenetic relationship between prohibitin and IgG4-RD.

### 2.9. Antibodies to Annexin A11

Hubers et al. used the human cholangiocyte cell line H69 to screen the serum samples of 50 patients with AIP or IgG4-associated cholangitis (IAC) [25]. A 56 kDa protein band was detected using Western blot analysis. Sera from 18% (9/50) of patients with AIP or IAC reacted to this band. However, reactivity was not detected with sera from patients with PSC or pancreatobiliary malignancies. This protein was identified as the calcium-dependent phospholipid-binding protein annexin A11 using affinity purification and mass spectrometry analysis [25]. ELISA analysis showed that two epitopes on the N-terminal domain of annexin A11 are shared between patients with AIP and those with IAC. Notably, the epitopes are recognized, not only by IgG4 but also by IgG1. The binding of IgG1 is inhibited by IgG4, which suggests that the levels of IgG4 could be elevated, resulting in the suppression of IgG1 immune response [25]. The frequency of annexin A11 autoantibody is low, and the role of annexin A11 in the pathogenesis of AIP remains unclear; therefore, further research is warranted.

### 2.10. Antibodies to Laminin 511-E8

**Figure 2:**
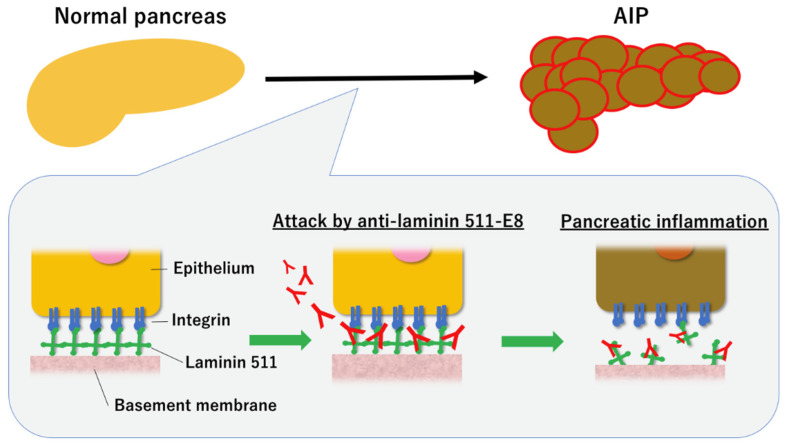
Proposed pathogenetic mechanism of autoimmune pancreatitis (AIP). The binding of specific pathogenetic autoantibodies to the basement membrane causes pancreatic inflammation, leading to AIP.

Laminin-511 is a heterotrimer that constitutes the extracellular matrix (ECM) of the pancreas [45], with laminin 511-E8 being a truncated form of laminin 511 [46]. We identified laminin 511-E8 as a pathological autoantigen in AIP [15]. We initially injected IgG of a patient with AIP subcutaneously into neonatal male BALB/c mice to investigate the pathophysiology of the circulating IgG of patients with AIP [10]. Pancreatic and salivary gland injuries are induced in mice injected with IgG of patients with AIP but not in control mice. Tissue-staining revealed that the IgG of patients with AIP binds to the base of the acini and interlobular space of the pancreatic tissues (Figure 2) [10]. IgG1 of patients with AIP is more pathogenic than IgG4. However, IgG4 significantly attenuates the pathogenic effect of IgG1 when injected simultaneously, suggesting a regulatory role for IgG4. These findings indicate that the patients with AIP have pathological autoantibodies specific to the basement membrane or the ECM of pancreatic tissue [10]. Therefore, we identified laminin 511-E8 as a target autoantigen in AIP [15]. Antibodies against laminin 511-E8 were detected using ELISA in 51% (26/51) of patients with AIP but only 1.6% (2/122) of the controls. Immunization with human laminin 511-E8 induces AIP-like lesions [15]. However, larger studies are required for investigating whether patients with AIP in western countries also have antibodies to laminin 511-E8 because all the patients in our study were Japanese. Further studies are also required for verifying whether these antibodies could be useful for the diagnosis of AIP.

### 2.11. Interferon (IFN)-α and Interleukin (IL)-33

Minaga et al. reported that IFN-α and IL-33 may serve as biomarkers for AIP and IgG4-RD [26]. They previously reported that IFN-α and IL-33 produced by plasmacytoid dendritic cells (pDCs) mediate chronic fibro-inflammation in both experimental AIP and human type 1 AIP [47]. Pancreatic accumulation of pDC is associated with the immunopathogenesis of type 1 AIP and IgG4-RD. In a case of type 1 AIP and IgG4-RD, the serum levels of IFN-α and IL-33 correlated better with disease activity than that of IgG4 [48]. They hypothesized that IFN-α and IL-33 may be potential biomarkers for type 1 AIP and IgG4-RD. The usefulness of IFN-α and IL-33 as biomarkers for AIP/IgG4 was evaluated in 21 patients with AIP/IgG4, 12 patients with chronic pancreatitis, and 8 healthy subjects. The sensitivity of IFN-α and IL-33 were 85.7% each, and their specificities were 91.7% and 83.3%, respectively [26]. Both IFN and IL-33 exhibited a strong correlation with IgG4 [26]. However, this study was a single-center retrospective study; a large multicenter study is needed to verify their findings.

### 2.12. Long non-Coding RNAs (lncRNAs)

lncRNAs are RNAs longer than 200 nucleotides that are not translated into proteins [49]. Initially, lncRNAs were recognized as non-functional junk RNAs but are now found associated with various diseases, such as malignancies, chronic inflammatory disorders, and autoimmune diseases [50,51,52]. Therefore, lncRNAs could be useful biomarkers for the diagnosis of AIP. Some lncRNAs, such as uc.308-, BC158811, BC166549, BC166474, and BC161988, could be potential biomarkers for diagnosis and promising targets for treating acute pancreatitis [53]. Further studies are needed to verify that these potential biomarkers are useful for the differentiators of AIP.

## 3. Predictive Biomarkers for AIP Relapse

The majority of the patients with AIP respond well to steroid therapy; however, relapse occurs often, even after remission with steroid therapy. The usefulness of MST for preventing AIP relapse has been controversial, but some reports in Japan support using MST following the remission of AIP [12,54]. Kamisawa et al. reported that the recurrence rate of AIP was significantly lower in patients undergoing MST (23.1%) compared to that in patients not receiving MST (33.7%) [12]. Masamune et al. performed a randomized controlled trial to ascertain the usefulness of MST in AIP following remission with steroid treatment [54]. They reported a relapse in 57.9% of patients not receiving MST within 3 years, while only 23.3% of the patients receiving MST relapsed [54]. However, the appropriate therapy and follow-up management of AIP following remission is not established. The presence of diffuse pancreatic swelling and IAC-complicated AIP are considered clinical predictors of AIP relapse; however, objective evaluation using biomarkers is desirable [13]. Serum levels of some candidate biomarkers decline following steroid treatment, suggesting their use in monitoring steroid treatment [15,20,21,26]. However, there are few biomarkers associated with the prediction of AIP relapse. Here, we review the potential predictive biomarkers of AIP relapse.

### 3.1. IgG4

IgG4 may be useful, not only in diagnosis but also for predicting relapse of AIP. Kubota et al. conducted a study on 70 patients with AIP to identify predictive factors associated with relapse. A high level of IgG4 (>135 mg/dL) during diagnosis is associated with an increased risk of relapse [55]. Suzuki et al. reported that patients with AIP, whose IgG4 levels re-elevate following steroid treatment are more likely to relapse than those without re-elevated levels [56]. Kamisawa et al. reported that patients with AIP, who maintain high levels of serum IgG4, even after steroid treatment, are more likely to relapse (29.6%) compared to those with normal serum IgG4 levels (10.1%) [12]. It is essential to establish a cutoff value of IgG4 levels for predicting AIP relapse.

### 3.2. Autotaxin

Fukiage et al. suggested serum autotaxin (ATX) as a possible biomarker for predicting relapse of AIP [57]. ATX is a secreted enzyme involved in the production of lysophosphatidic acid (LPA) [58]. LPA mediates signals related to cell survival, migration, and proliferation via G protein-coupled receptors [59]. Fukiage and colleagues reported that the level of ATX decreases during treatment, to establish remission, and during maintenance therapy, compared to that before treatment. In patients who relapse, serum ATX levels significantly increase at the time of relapse, compared to that during induction therapy (*p* = 0.039). ATX may be a useful biomarker, not only for predicting AIP relapse, but also for monitoring the progress of steroid therapy [57]. However, the study by Fukiage et al. focused on male patients with AIP, because the serum levels of ATX are significantly higher in women, compared to that in men [60,61]. Further studies that include female patients are required to ascertain the usefulness of ATX as a biomarker.

## 4. New Candidate Biomarkers for Diagnosis of IgG4-RD

Antibodies to Galectin-3

Perugino et al. attempted to identify the antigen for IgG4-RD using various methodologies, such as next-generation sequencing, single-cell cloning of dominantly expanded plasmablasts, monoclonal antibody production, antigen purification, and mass spectrometry [62]. Galectin-3 was identified as a potential autoantigen in patients with IgG4-RD. ELISA analysis revealed that anti-galectin-3-specific IgG4 antibody was present in 28.1% (34/121) of the patients with IgG4-RD but was not detected in the controls [62]. Galectin-3 is a member of the β-galactoside-binding lectin family and plays an important role in the pathogenesis of fibrotic disorders in multiple organs, including the lung, kidney, heart, and liver [63]. Inhibition of galectin-3 mitigates bleomycin-induced pulmonary fibrosis in mouse models; and therefore, it could be an effective therapeutic target in these disorders [64,65].

The autoantibody response to prohibitin, annexin A11, laminin 511-E8, and galectin-3 in patients with IgG4-RD (*n* = 100) were studied [66]. Among them, 10% of patients with IgG4-RD are positive for prohibitin, 12% are positive for annexin A11, 7% are positive for laminin 511-E8, and 25% are positive for galectine-3. Approximately 14% of the patients show autoantibody responses to two or more of these four autoantigens. These patients have higher levels of the IgG subclasses, exhibit a greater incidence of hypocomplementemic, and more frequent involvement of other organs compared to that in other patients [66]. Antibodies to laminin 511-E8 in patients with AIP and antibodies to annexin A11 in patients with AIP/IAC are reported, and therefore, the response frequencies of anti-laminin 511-E8 and anti-annexin A11 antibodies in IgG4-RD patients with and without pancreatic or biliary involvement were compared [15,25,66]; No significant differences were observed [66]. The reason for the differential response frequencies of antibodies, other than that for galectine-3, is unclear [15,24,25]. One possible explanation is the genetic differences between the cohorts studied [66,67].

## 5. Conclusions

Recent findings on the potential biomarkers of AIP were described in this review. A number of possible biomarkers have been reported for AIP, but unfortunately, none of these biomarkers is specific for AIP. Further elucidation of the pathogenesis of AIP may lead to the discovery of a dominant autoantibody. Additional studies are warranted for establishing a diagnostic biomarker for AIP.

## Figures and Tables

**Table 1:** Candidate biomarker for the diagnosis of autoimmune pancreatitis.

No.	Candidate Biomarker	Positivity in AIP (%) (No. Positive/No. Tested)	Positivity in Controls (%) (No. Positive/No. Tested)	Screening Method	Reference
**1**	IgG4	95 (19/20) 75.6 (34/45)	2.9 (2/70) pancreatic cancer 9.6 (13/135) pancreatic cancer	Single radial immunodiffusion Automated nephelometry	[3,16]
**2**	Anti-carbonic anhydrase II	33.3 (11/33)	61.9 (13/21) Sjögren’s syndrome 15 (3/20) alcoholic pancreatitis 0 (0/7) cholecytic pancreatitis	ELISA	[17]
**3**	Anti-lactoferrin	76.5 (13/17)	0 (0/51)	ELISA	[18]
**4**	Anti-pancreatic secretory trypsin inhibitor	42.3 (11/26) on Western blotting 30.8 (8/26) on ELISA	0 (0/65) on ELISA	Western blotting ELISA	[19]
**5**	Anti-amylase-2A	100 (15/15)	88.2 (15/17) fulminant type 1 diabetes 21.4 (9/42) acute type1 diabetes 6 (4/67) type 2 diabetes 1 (1/100) normal control	ELISA	[20]
**6**	Anti-heat shock protein	91.7 (11/12)	81.3 (13/16) fulminant type 1 diabetes 8.3 (2/24) chronic alcoholic pancreatitis 1.4 (1/71) normal control	ELISA	[21]
**7**	Anti-trypsinogen	79 (sensitivity)	5 (1-specificity)	ELISA	[22]
**8**	Anti-plasminogen-binding protein	95 (19/20)	10 (4/40) pancreatic cancer 0 (0/76) other controls	DELFIA	[23]
**9**	Anti-prohibitin	73.5 (25/34)	53.3 (8/15) Mikulicz’s disease 54.5 (6/11) retroperitoneal fibrosis 89.7 (26/29) other probable IgG4-RD 1.4 (1/70) normal control	ELISA	[24]
**10**	Anti-annexin A11	18 (9/50) (including IAC)	0 (0/20) primary sclerosing cholangitis 0 (0/27) pancreatic cancer	Western blotting	[25]
**11**	Anti-laminin 511-E8	51 (26/51)	1.6 (2/122) healthy control and disease control	ELISA	[15]
**12**	IFN-α	85.7 (sensitivity)	8.3 (1-specificity)	ELISA	[26]
**13**	IL-33	85.7 (sensitivity)	6.7 (1-specificity)	ELISA	[26]

AIP: autoimmune pancreatitis, ELISA: enzyme-linked immunosorbent assay, DELFIA: dissociation-enhanced lanthanide fluorescence immunoassay, IgG4-RD: IgG4-related disease, IAC: IgG4-associated cholangitis, IFN: interferon, and IL: interleukin.

## Data Availability

Not applicable.
